# Enhancing Antimicrobial Efficacy and Synergistic Effects of Nano-Silica-Based Combinations With Doxycycline, Metronidazole, and Ciprofloxacin Against Enterococcus faecalis Biofilms

**DOI:** 10.7759/cureus.54668

**Published:** 2024-02-22

**Authors:** Shahul Hameed, Delphine P Antony, Rajeshkumar Shanmugam, Sandhya Raghu, Hima Sandeep Adimulapu

**Affiliations:** 1 Conservative Dentistry and Endodontics, Saveetha Dental College and Hospital, Saveetha Institute of Medical and Technical Sciences, Saveetha University, Chennai, IND; 2 Nanobiomedicine Lab, Centre for Global Health Research, Saveetha Medical College and Hospital, Saveetha Institute of Medical and Technical Sciences, Chennai, IND; 3 Conservative Dentistry and Endodontics, Saveetha Dental College and Hospital, Saveetha Institute of Medical and Technical Sciences, Chennai, IND

**Keywords:** biofilm, silica nanoparticles, e.faecalis, agar well diffusion technique, antimicrobial efficacy

## Abstract

Background: *Enterococcus faecalis *biofilm formation within root canals poses a challenging problem in endodontics, often leading to treatment failure. To combat this issue, nanotechnology offers a promising avenue for enhancing antimicrobial efficacy. This study explores the potential synergistic effects of combining nanoscale silica particles with conventional antibiotics, including doxycycline, metronidazole, and ciprofloxacin, against *E. faecalis* biofilms. The unique characteristics of silica nanoparticles, such as their increased reactivity and ability to be functionalized with other compounds, make them ideal candidates for augmenting antibiotic efficacy. This research investigates the antimicrobial properties of these silica-based combinations and their potential to eliminate or inhibit *E. faecalis* biofilms more effectively than conventional treatments.

Methodology: The methods involved the preparation of nanostructured silica particles and their combination with doxycycline, Flagyl, and ciprofloxacin at subinhibitory concentrations. These combinations were then tested against *E. faecalis* biofilms using the agar well diffusion technique.

Results: Preliminary results suggested that the synergistic interactions between silica nanoparticles and antibiotics can significantly enhance antimicrobial efficacy. The combined treatment exhibited superior inhibitory effects on *E. faecalis* compared to antibiotics or silica nanoparticles alone (*P *< 0.05).

Conclusions: This study sheds light on the potential of nanoscale silica-based combinations to address the challenges posed by *E. faecalis* biofilms in endodontics. Understanding the mechanisms of synergy between nanoparticles and antibiotics can pave the way for the development of more effective and targeted strategies for root canal disinfection, ultimately improving the success rates of endodontic treatments.

## Introduction

Nanotechnology plays a significant role in the field of endodontics, leveraging the unique properties of nanosized particles, including small clusters of atoms or molecules, to enhance various aspects of endodontic treatment [1]. One of the notable applications of nanotechnology in endodontics is the modification of rotary nickel-titanium files at the nanoscale, which leads to improved resistance against wear and fatigue [2]. Nanoparticles (NPs) have a broad range of potential uses in endodontics, particularly in enhancing the properties of various materials used in the treatment process. This includes improving the effectiveness of irrigants, medicaments, sealers, and obturating materials, which ultimately enhances the disinfection and sealing of the root canal system [3]. In the field of regenerative endodontics, nanobiomaterials are employed to create advanced scaffolds and facilitate the controlled delivery of growth factors, contributing to more successful regenerative procedures [4]. Furthermore, NPs are being investigated for their antibacterial properties, to eliminate biofilms and bacteria within the root canal system [5].

Biofilm development by *Enterococcus faecalis* in the field of endodontics poses a notable concern and represents a significant factor leading to the failure of root canal treatments [6]. Traditional approaches used to manage bacterial biofilm infections within root canals have demonstrated limited effectiveness, resulting in recurrent and persistent infections that are difficult to treat [7]. Compounding the issue is the fact that *E. faecalis* can create biofilms even when exposed to suboptimal antibiotic concentrations, leading to increased resistance and reduced susceptibility to antibiotics [8]. These biofilms formed by *E. faecalis* can be particularly troublesome and are responsible for enduring endodontic infections [9]. Fortunately, research has shown that propolis NPs exhibit promising antibacterial properties against *E. faecalis* biofilms within the root canal system of endodontics [10]. 

Silica NPs offer promising prospects in the field of endodontics. They have found utility in various applications, including drug delivery systems, serving as intracanal medication, and being employed alongside sealers or restorative materials to enhance antimicrobial effectiveness in both endodontic and conservative treatments [11]. These NPs possess distinct characteristics attributable to their minuscule size, such as increased antibacterial potency, enhanced reactivity, and the ability to combine with other substances [4]. Researchers have explored their use as irrigants and intracanal medicaments to improve the eradication of endodontic infections, including bacterial biofilms [12]. Moreover, silica NPs have been investigated for their potential role in regenerative endodontics, aiding in the controlled release of bioactive compounds and improving scaffold properties [13]. Clinical trials have provided evidence of the safety, efficacy, and suitability of silica NPs in various biomedical applications, including oral drug delivery [14].

The triple antibiotic paste (TAP) is a frequently employed substance in endodontic root canal treatments. It comprises ciprofloxacin, metronidazole, and minocycline, all of which possess antibacterial properties contributing to the success of root canal therapy. TAP is highly effective in disinfecting the root canal and inhibiting bacterial growth by impeding processes such as DNA replication and protein synthesis [15]. This medication serves as an alternative to calcium hydroxide, the most commonly used intracanal medicament [16]. Notably, TAP demonstrates effectiveness against resilient microorganisms like* E. faecalis* and *Candida albicans*, which are often implicated in root canal infections [17]. However, it's essential to be aware that the use of TAP has been linked to tooth discoloration [18]. 

## Materials and methods

Synthesis of nano-silica

In a sterile conical flask, a combination of 1.57 mL of ammonia (as the solvent), 37 mL of ethanol (also a solvent), and 5 mL of water was gently combined. After stirring the mixture for 5 minutes, an additional 3 mL of tetraethoxysilane (TEOS) was introduced, and the stirring process continued for an hour. Subsequently, the silica NPs were separated by subjecting the mixture to centrifugation at 10,000 rpm for 30 minutes. The resulting pellet was then dried using a hot air oven at a temperature of 60 °C. Analyses such as X-ray diffraction (XRD), energy-dispersive X-ray spectroscopy (EDS), Fourier transform infrared spectroscopy (FTIR), scanning electron microscopy (SEM), and transmission electron microscopy (TEM) were conducted on the same samples.

Preparation of silica NP-based antibiotic combinations

Silica NPs were combined with antibiotics, including doxycycline (100 mg), metronidazole (100 mg), and ciprofloxacin (100 mg), in a 1:1 ratio, respectively. Subsequently, these combinations were dissolved in 1 mL of distilled water and subjected to mixing on a vortex mixer for 10-15 minutes. This procedure was conducted to prepare the nano-silica-based antibiotic combinations.

Antimicrobial activity

The bacterial strains (*E. faecalis* ATCC29212), subcultured for 24 hours, were transferred to a laminar flow hood to prepare bacterial suspensions. The turbidity of these suspensions was compared to a 0.5% MacFarland standard solution. Following this, autoclaved cotton swabs were immersed in the suspensions and then used to streak the surface of Mueller-Hinton agar (MHA) plates, creating a uniform bacterial lawn culture. Next, a sterile cork borer was employed to create three evenly spaced wells within three separate quadrants of each MHA plate. Each well was meticulously labeled and subsequently filled with a pipetted solution of 30, 60, and 90 µg/mL of silica NPs, a combination of NPs and doxycycline, a combination of NPs and metronidazole, a combination of NPs and ciprofloxacin, and finally, a combination of NPs, doxycycline, metronidazole, and ciprofloxacin from the prepared solution as mentioned in the preparation protocol. The MHA plates were then incubated at 37 °C for 24 hours, and the results were recorded the following day. To ensure accuracy, each test was repeated three times to obtain an average value for the zones of inhibition. 

Statistical analysis

The collected data were analyzed and presented. One-way analysis of variance (ANOVA) and post hoc tests were used to assess the statistical significance when comparing within-group and between-group differences using IBM SPSS Statistics for Windows, Version 23.0 (IBM Corp., Armonk, NY) (*P *< 0.05).

The groups were as follows: Group 1, silica NP; Group 2, NP + doxycycline; Group 3, NP + metronidazole; Group 4, NP + ciprofloxacin; Group 5, NP + doxycycline + metronidazole + ciprofloxacin.

## Results

Bernardi et al. stated that antibiotic compounds together with functionalization were required to enhance the effect against resistant bacteria [8]. Taking this into consideration, NPs were included in this study. The antimicrobial activity of NP against *E. faecalis* was assessed at different concentrations of the NPs and in combination with various antibiotics, including doxycycline (doxy), metronidazole (Flagyl), and ciprofloxacin (Cipro). 

**Figure 1:**
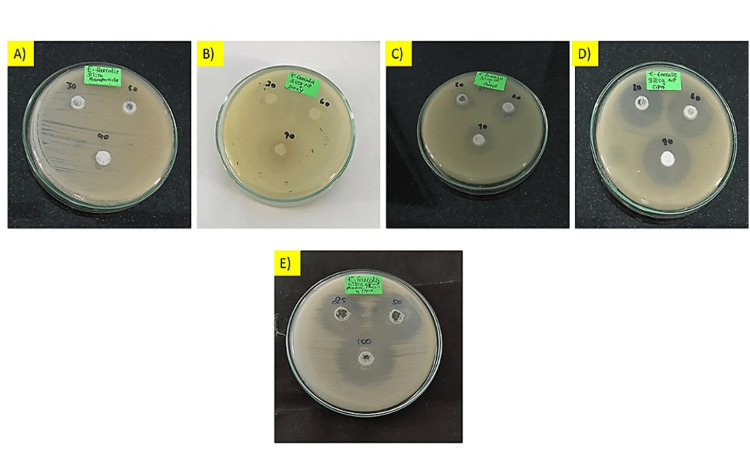
Antimicrobial activity of silica nanoparticle and combination of antibiotics: (A) silica nanoparticle (NP); (B) NP + doxycycline; (C) NP + metronidazole; (D) NP + ciprofloxacin; (E) NP + doxycycline + metronidazole + ciprofloxacin.

The antimicrobial activity was assessed by measuring the inhibition of *E. faecalis* growth in the presence of different treatments (Figure 1). The antimicrobial activity of the silica NP against *E. faecalis* showed a concentration-dependent effect. As the volume of NP increased from 30 to 90 µg/mL, there was a corresponding increase in antimicrobial activity. At the highest concentration (90 µg/mL), NP exhibited the highest inhibitory effect, with a zone of inhibition showed 16 mm. When NP was combined with doxycycline (NP + doxy), the antimicrobial activity increased compared to NP alone. At the highest concentration (90 µg/mL), NP + doxy showed a zone of inhibition of 20 mm.

**Figure 2:**
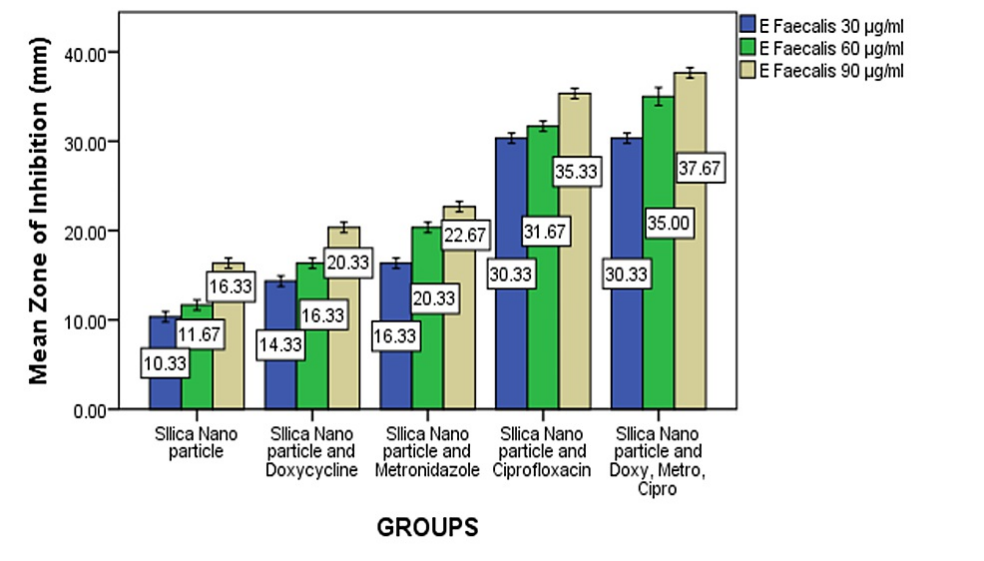
Antimicrobial efficacy of silica nanoparticles and their antibiotic combinations against Enterococcus faecalis.

Similarly, the combination of NP with metronidazole (NP + metronidazole) and ciprofloxacin (NP + Cipro) also enhanced antimicrobial activity. At the highest concentration (90 µg/mL), NP + metronidazole exhibited a zone of inhibition up to 23 mm, while NP + Cipro showed an inhibition of 35 mm (Figure 2). The most significant enhancement in antimicrobial activity was observed when all three antibiotics, doxycycline, metronidazole, and ciprofloxacin, were combined with NP (NP + doxy + metro+ Cipro). At the highest concentration (90 µg/mL), this combination resulted in a substantial growth inhibition of 38 mm. To test the significance between the groups, one-way ANOVA and post hoc Tukey analyses were conducted based on the obtained values. The results indicated a significant difference at concentrations of 30, 60, and 90 µg/mL. All three concentrations of all the groups showed significant differences from each other (*P *< 0.05). Group 5 showed more zones of inhibition than other groups (*P *< 0.05) (Table 1).

**Table 1:** Mean and standard deviation for the zone of inhibition of all the groups. Along the column, different superscript uppercase letters show statistically significant differences (*P *< 0.05). Along the row, different superscript lowercase letters show statistically significant differences (*P *< 0.05).

Groups	*Enterococcus faecalis* (30 µg/mL)	*E. faecalis* (60 µg/mL)	*E. faecalis* (90 µg/mL)
Silica nanoparticle	10.3333 ± 0.57735^A,a^	11.6667 ± 0.57735^A,a^	16.3333 ± 0.57735^A,b^
Silica nanoparticle and doxycycline	14.3333 ± 0.57735^B,a^	16.3333 ± 0.57735^B,a^	20.3333 ± 0.57735^B,b^
Silica nanoparticle and metronidazole	16.3333 ± 0.57735^C,a^	20.3333 ± 0.57735^C,a^	22.6667 ± 0.57735^C,b^
Silica nanoparticle and ciprofloxacin	30.3333 ± 0.57735^D,a^	31.6667 ± 0.57735^D,a^	35.3333 ± 0.57735^D,b^
Silica nanoparticle and doxycycline, metronidazole, ciprofloxacin	30.3333 ± 0.57735^E,a^	35.0000 ± 1.00000^E,a^	37.6667 ± 0.57735^E,b^

## Discussion

Nanosilica has been investigated for its potential antimicrobial efficacy against *E. faecalis* [19]. In a comparative study evaluating the effectiveness of different intracanal medicaments, it was observed that nanosized calcium hydroxide (CH) and chlorhexidine (CHX) exhibited superior antimicrobial properties compared to their conventional-sized counterparts in eradicating *E. faecalis* biofilm formations [20]. Ghahramani et al. stated that a combination of antimicrobial agents used as intra-canal medicament was better compared to a single agent [20].

Furthermore, a study conducted to assess the antimicrobial efficacy of CH and TAP against *E. faecalis* in infected primary molars revealed significant antimicrobial effects for both medications [20]. One study conducted an assessment of the antimicrobial effectiveness of a TAP consisting of ciprofloxacin, metronidazole, and cefaclor, in combination with chitosan and CH, and compared it with a control using normal saline. The study concluded that the paste containing chitosan exhibited the highest level of antimicrobial efficacy against *E. faecalis* [21]. 

In another investigation, the antibacterial properties of biopolymer-coated ceramic microparticles loaded with a modified combination of three antibiotics (Penicillin G, metronidazole, and ciprofloxacin) were evaluated in the context of combating *E. faecalis*. The results indicated that these microparticles were capable of producing the largest zone of growth inhibition [22]. These findings collectively emphasize the potential of innovative approaches involving antibiotic combinations and biopolymer-coated materials for enhancing antimicrobial effects against *E. faecalis* in dental applications.

The combination of NPs with antibiotics presents a promising strategy in the battle against periodontal pathogens, particularly *E. faecalis*, a contributor to periodontitis. Metal NPs like silver (AgNPs), gold (AuNPs), and copper (CuNPs) exhibit potent antibacterial properties against a broad spectrum of bacteria, including those resistant to traditional antibiotics [23]. This synergistic approach offers multiple benefits, including enhanced antibacterial activity, leading to a more substantial reduction in bacterial populations. Furthermore, NPs can circumvent bacterial resistance mechanisms, making it challenging for bacteria to develop resistance [24]. Additionally, the targeted drug delivery capability of NPs allows for selective targeting of bacterial cells, minimizing damage to healthy cells and reducing systemic side effects. Moreover, engineered NPs enable a sustained release of antibiotics over an extended period, ensuring a continuous and localized supply of antibiotics at the infection site [25]. 

The highest inhibition was achieved when all three antibiotics were used in conjunction with NPs, suggesting a potential strategy for improving the effectiveness of antimicrobial treatments against infections caused by *E. faecalis.* The limitation of the study was that other organisms were to be tested to check for the efficacy of the combination of silica NPs with antibiotics. Further, checking its effectiveness in clinical scenarios requires additional exploration.

## Conclusions

The synergistic effects of combining nanoscale silica particles with conventional antibiotics, including doxycycline, metronidazole, and ciprofloxacin, against *E. faecalis *holds promise for addressing a persistent challenge in endodontics. *E. faecalis* biofilm formation within root canals often leads to treatment failures, necessitating innovative approaches for enhanced antimicrobial efficacy. The unique properties of silica NPs, characterized by their heightened reactivity and amenability to functionalization, have been harnessed in this study to augment the performance of antibiotics. Ultimately, these findings offer hope for improving the success rates of endodontic treatments, as the enhanced antimicrobial properties of silica-based combinations may prove instrumental in addressing the challenge posed by *E. faecalis* biofilms within root canals. This research opens the door to innovative approaches in endodontics, with the potential to elevate patient outcomes and contribute to the long-term success of root canal therapies.
